# Phase I study of docetaxel plus ifosfamide in patients with advanced cancer

**DOI:** 10.1038/sj.bjc.6600542

**Published:** 2002-10-07

**Authors:** G Marx, C Lewis, K Hall, J Levi, S Ackland

**Affiliations:** Department of Medical Oncology, Prince of Wales Hospital, Sydney, Australia; Newcastle Mater Misericordiae Hospital, NSW, Australia; Department of Medical Oncology, Royal North Shore Hospital, Sydney, NSW, Australia

**Keywords:** ifosfamide, docetaxel, malignancy, toxicity, G-CSF

## Abstract

The aim of this study was to determine the maximum tolerated dose of a fixed dose of docetaxel when combined with continuous infusion ifosfamide, with and without G-CSF support, in the treatment of advanced cancer, and to evaluate anti-tumour activity of this combination. Thirty-one patients with advanced malignancies were treated with docetaxel 75 mg/m^2^ intravenously on days 1, and ifosfamide at increasing dose levels from 1500 mg/m^2^/day to 2750 mg/m^2^/day as a continuous infusion from day 1–3, every 3 weeks. A total of 107 cycles of treatment were administered. Without G-CSF support dose-limiting toxicity of grade 4 neutropenia greater than 5 days duration occurred at dose level 1. With the addition of G-CSF the maximum tolerated dose was docetaxel 75 mg/m^2^ on day 1 and ifosfamide 2750 mg/m^2^/day on days 1–3. Dose limiting toxicity (DLT) included ifosfamide-induced encephalopathy, febrile neutropenia and grade three mucositis. Three complete responses and 3 partial responses were seen. This combination of docetaxel and infusional ifosfamide is feasible and effective. The recommended dose for future phase II studies is docetaxel 75 mg/m^2^ on day 1 and ifosfamide 2500 mg/m^2^/day continuous infusion on days 1–3.

*British Journal of Cancer* (2002) **87**, 846–849. doi:10.1038/sj.bjc.6600542 www.bjcancer.com

© 2002 Cancer Research UK

## 

Docetaxel and ifosfamide have both demonstrated significant single agent anti-tumour activity in a number of tumours. This phase I study was performed to assess the feasibility, toxicity and maximum tolerated dose (MTD) of this combination.

Ifosfamide is an alkylating agent with activity in a variety of solid tumours, including non-small cell lung cancer (NSCLC), sarcoma, testicular cancer, breast cancer and lymphoma (Pinedo and Verweij, 1986; Shirinian *et al*, 1992; Kaye *et al*, 1998; Moskowitz *et al*, 1999). The major toxicities of ifosfamide include myelosuppression and haemorrhagic cystitis. The incidence of cystitis can be reduced with co-administration of mesna, which functions as a regional detoxicant of acrolein, the urotoxic metabolite of ifosfamide. Other side effects include nephrotoxicity, encephalopathy, nausea, vomiting and alopecia. Encephalopathy appears to be more common in patients receiving oral or bolus ifosfamide and in patients with low serum albumin, low serum potassium or renal dysfunction (Meanwell *et al*, 1986; Watkin *et al*, 1989; Cerny *et al*, 1990).

The optimum dosing schedule for ifosfamide is still uncertain. The pharmacokinetics of ifosfamide is schedule dependent, but large inter-patient variability in ifosfamide pharmacokinetics and metabolism is seen (Lind *et al*, 1990). It has been administered orally or intravenously as bolus injection, short-term or continuous infusion, and either in 1 day, or in divided doses over several days. In this study ifosfamide was administered as a continuous infusion over 3 days, as this schedule has been demonstrated to have a lower incidence of haemorrhagic cystitis and neutropenia (Lind *et al*, 1990).

Docetaxel is a semi-synthetic taxane with clinical activity in a wide range of tumours (Pazdur *et al*, 1992; Ravdin *et al*, 1995; Kavanagh *et al*, 1996; Roth *et al*, 2000; Shepherd *et al*, 2000; Kaye 2001; Nabholtz *et al*, 2001; Aihara *et al*, 2002; Smith *et al*, 2002). The major dose limiting toxicity (DLT) is myelosuppression, predominantly neutropenia, which is usually short lasting, dose dependent and non-cumulative (Extra *et al*, 1993; Tomiak *et al*, 1994; Pronk *et al*, 1998). Other toxicities are usually mild and easily managed or prevented. Fluid retention is a significant and well-documented cumulative side effect of docetaxel, which is reduced by pre-medication with corticosteroids (Bizzari and Bail 1994). A fixed dose of docetaxel was chosen because of concerns regarding the toxicities of fatigue, neuropathy and fluid retention at doses >75 mg/m^2^.

Although the DLT for both drugs is neutropenia, the absence of other significant overlapping toxicity and the availability of G-CSF support renders this combination a potentially useful therapy for a variety of advanced cancers.

The objectives of this phase I study were to identify the MTD of docetaxel plus ifosfamide with and without G-CSF support, and to characterize the toxicity profile of the combination.

## PATIENTS AND METHODS

### Patient selection

Patients were enrolled onto this study if they met the following criteria: histologically confirmed advanced cancer, for which other forms of therapy had failed or were considered inappropriate; no more than one previous line of chemotherapy, >4 weeks prior to enrollment; no prior exposure to a taxane or ifosfamide. Other eligibility criteria included age ⩾17 years; ECOG performance status ⩽2; expected survival duration of ⩾3 months; adequate bone marrow function (absolute neutrophil count ⩾2.0×10^9^/l, platelets ⩾100×10^9^/l and Hb ⩾100 g/l); adequate hepatic function (total bilirubin <1×upper limit of normal (ULN); AST and ALT ⩽2.5×ULN, ALP ⩽5×ULN (except in presence of bone only metastases and other liver function tests normal, if both AST and/or ALT ⩾1.5×ULN and ALP ⩾2.5×ULN patients were excluded)); adequate renal function (serum creatinine ⩽1.5×ULN). Patients were excluded with significant co-morbid medical conditions; symptomatic peripheral neuropathy >grade 2 according to NCIC Common Toxicity Criteria; and presence of CNS disease. The Research Ethics Committee at each participating institution approved the study. Written informed consent was obtained from all patients prior to study entry.

### Drug administration

Docetaxel (Taxotere; RP56976) was supplied in vials by Rhone-Poulenc Rorer as a concentrated sterile solution containing 40 mg in 1 ml or 80 mg in 2 ml polysorbate 80. The appropriate amount of drug was diluted in 500 ml of 5% dextrose and administered as a 1-hour infusion at a fixed dose of 75 mg/m^2^ on day 1.

Ifosfamide was diluted in 3 litres of dextrose–saline with mesna at an equimolar dose and administered as a 24-h continuous infusion daily for 3 days, commencing immediately after completion of docetaxel. An intravenous bolus dose of 500 mg mesna was given immediately prior to commencing the ifosfamide infusion on day 1, and at completion of the treatment on day 3. Treatment cycles were repeated every 3 weeks.

Premedication with dexamethasone orally at a dose of 8 mg at 13, 7, and 1 h prior to treatment and then at 12, 24, and 36 h post therapy. Anti-emetics were administered as per physician and institution preference. These usually included a 5HT_3_ antagonist.

Granulocyte-colony stimulating factor (G-CSF, Granocyte®, Amrad Australia) was administered at a dose 150 μg/m^2^/day (=1 vial daily) to all patients prophylactically after all three patients entered on level 1A experienced grade 4 neutropenia greater than 5 days duration during the first cycle of treatment. G-CSF was commenced on day 4 and continued until the absolute neutrophil count was ⩾10×10^9^/l at all other dose levels.

### Dosage and dose escalation procedure

Docetaxel dose was fixed at 75 mg/m^2^, and ifosfamide dose was escalated in groups of at least three patients according to a pre-defined schedule until the MTD was identified (see Table 2

**Table 2:**
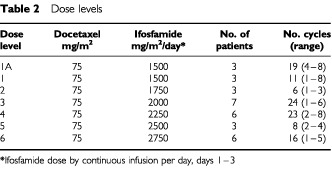
Dose levels

). Dose escalation within patients was not permitted. Three patients were recruited at each dose level. Before escalating to the next dose level at least three patients should have received one cycle and been observed for toxicity for 2 weeks. If one out of three patients at any dose level developed a DLT, three more patients were entered at that dose level. The MTD was defined as the dose at which three or more out of six patients developed a dose limiting toxicity. Dose limiting toxicity was defined as febrile neutropenia, grade 4 neutropenia greater than 5 days duration, grade 4 thrombocytopenia, and any grade 3 potentially life threatening toxicity occurring in cycle 1. The intention of the study was to treat at full doses at each dose level and avoid dose reductions. If patients experienced significant toxicity other than the defined dose limiting events, treatment was delayed until recovery to grade 0–1, and then restarted, for the subsequent cycles, at the dose level below. All toxicity data was reported as per the initial dose level. Toxicities were graded according to the NCIC Common Toxicity Criteria.

### Evaluation

Pre-treatment evaluation included a full medical history and physical examination, complete blood count, differential white blood cell (WBC), biochemistry tests (serum electrolytes, renal function, hepatic function, calcium, phosphate, glucose), electrocardiogram and urinalysis. Disease assessment included routine chest radiograph, computerized tomography of chest, abdomen and pelvis, and radionuclide bone scan as clinically indicated within 3 weeks of commencing treatment.

Clinical review and serum biochemistry were repeated weekly during the first cycle of treatment and then every 3 weeks. A neurological examination was performed at baseline and at the beginning of each treatment cycle. Complete blood cell count was undertaken twice weekly. Urinalysis was performed before, during and after the ifosfamide infusion. Formal disease assessment was performed after every two cycles of treatment. Tumour response was classified according to standard World Health Organization criteria (Miller, *et al*, 1981).

## RESULTS

Thirty-one patients were entered into the study. All patients were evaluable for toxicity and 28 were evaluable for response. Patient characteristics are shown in Table 1

**Table 1:**
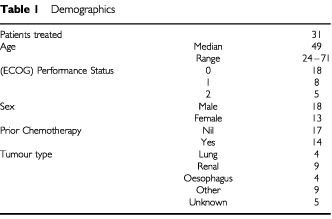
Demographics

. The median age was 49 years (range of 24–71). Eighteen patients were male and 13 were female. Seventeen patients had not received prior chemotherapy while the remainder had received one prior chemotherapy regimen. One hundred and seven cycles of treatment were given over six dose levels (median three cycles per patient, range 1–8, see Table 2). Dose limiting toxicity was reported in cycle 1 at the following dose levels: two episodes of encephalopathy at level 3; two episodes of febrile neutropenia at level 4; and two episodes of febrile neutropenia, two of encephalopathy, and one of mucositis at level 6 (docetaxel 75 mg/m^2^ with ifosfamide 2750 mg/m^2^/day), which was defined as the MTD.

### Haematological toxicity

Neutropenia grades 3 and 4 were experienced at all dose levels (Table 3

**Table 3:**
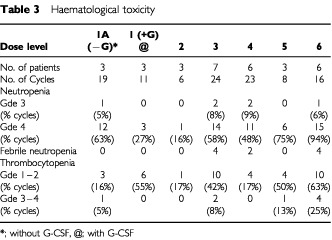
Haematological toxicity

). At level 1A, all three patients had grade 4 neutropenia lasting greater than 5 days, so prophylactic G-CSF was incorporated into the treatment of all future patients as planned. The severity of neutropenia was reduced at dose level 1, with G-CSF support, compared to without G-CSF support. At all higher dose levels the incidence of grade 3–4 neutropenia was ifosfamide dose dependent, but the duration of grade 4 neutropenia was less than 5 days. There were 10 episodes (9%) of febrile neutropenia but no septic deaths. Severe anaemia, grades 3 and 4, was seen in 17 cycles (16%). Grade 3–4 thrombocytopenia was uncommon, occurring in 7% of cycles at all dose levels.

### Non-haematological toxicity

Significant toxicities included nausea and vomiting, which was severe (grade 3–4) in only 6% of cycles (Table 4

**Table 4:**
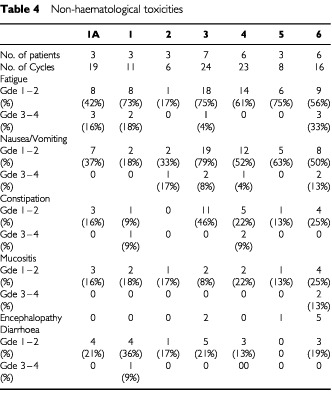
Non-haematological toxicities

). Six episodes resulted in admission for intravenous fluids. Alopecia was universal. Mucositis was generally mild with only two episodes of grade 3 mucositis at dose level 6. There were no episodes of docetaxel induced peripheral oedema. There were no episodes of grade 3 or 4 peripheral neuropathy. Most patients reported mild fatigue.

Ifosfamide-related encephalopathy occurred in eight patients, mainly at higher dose levels (level 3: two episodes, level 5: one episode, level 6: five episodes). Most episodes resolved spontaneously following cessation of the ifosfamide, but one patient required treatment with methylene blue. One episode was associated with deterioration in renal function but there was no correlation with patient hypoalbuminaemia or hypokalaemia.

### Responses

Twenty-eight of the 31 patients were assessable for response. One patient received only one cycle of treatment and ceased due to hepatic toxicity, compounded by the co-administration of ketoconazole. Complete response (CR) was achieved in three patients adenocarcinoma of oesophagus (duration of response 12 months), non-small cell lung cancer (18 months), and breast cancer (8 months). Three patients had a partial response adenocarcinoma of oesophagus (6 months), adenocarcinoma of unknown primary (7 months) and non-small cell lung cancer (3 months).

## DISCUSSION

In this study the MTD of the combination of docetaxel and ifosfamide was identified at 75 mg/m^2^ docetaxel on day 1 and 2750 mg/m^2^/day ifosfamide as a continuous infusion on days 1–3 with G-CSF cover. The dose limiting toxicities included encephalopathy, febrile neutropenia and mucositis.

The combination of docetaxel and ifosfamide is potentially effective in view of their non-cross resistant activity, and non-overlapping toxicity except for neutropenia. The major toxicity, as expected, was neutropenia, which was grade 3–4 neutropenia in 64% of cycles with 10 (9%) episodes of febrile neutropenia. Without G-CSF support, the MTD was reached at level 1A. The use of G-CSF allowed delivery of doses of each drug close to their single agent MTD, and significant non-haematological toxicity was then seen.

For this study we used a continuous infusion over three days because of a low reported incidence of haemorrhagic cystitis and myelosuppression compared with other schedules (Cerny *et al*, 1990; Lind *et al*, 1990). No instances of haemorrhagic cystitis were observed in this study but myelosuppression was common and dose-limiting as expected. We can speculate that other schedules of ifosfamide may also be combined with docetaxel but the appropriate dose in such circumstances is unclear. This protocol mandated a 3-day hospital admission, but administration using an outpatient-based drug infusion may be feasible.

In a previous phase I study of this combination, Pronk *et al* escalated the dose of both agents, and ifosfamide was infused over a 24-h period on day 1 only (Pronk *et al*, 1998). In addition they addressed the issue of scheduling, with docetaxel administered prior to ifosfamide in the first phase of the study, and reversal of the order of administration in the second part. When ifosfamide was given first, there was a higher incidence of gastrointestinal toxicity including vomiting and diarrhoea. As well, the DLT of neutropenic fever (common to both schedules) occurred at a lower dose when the ifosfamide was administered prior to docetaxel. The MTD occurred at docetaxel 85 mg/m^2^ and ifosfamide 5000 mg/m^2^, and the authors recommended that docetaxel be given prior to ifosfamide. In contrast to our study, there were no episodes of ifosfamide encephalopathy noted, and greater than 50% incidence of mild peripheral neuropathy, possibly due to a higher cumulative dose of docetaxel delivered (Pronk *et al*, 1998). It is difficult to compare different schedules of ifosfamide for relative efficacy or toxicity. However the total dose of ifosfamide deliverable in combination with 75 mg/m^2^ docetaxel is greater with a 3-day infusion (8500 mg/m^2^) than with a 24-hour infusion (5000 mg/m^2^).

Anti-tumour activity was seen at all dose levels. Of particular interest were the responses seen in two of four patients with adenocarcinoma of the oesophagus, and in two of four patients with non-small cell lung cancer.

In conclusion, the combination of docetaxel and infusional ifosfamide for patients with advanced cancer is tolerable and effective. The recommended dose for phase II studies is docetaxel 75 mg/m^2^ day 1 and ifosfamide 2500 mg/m^2^ on days 1–3 for previously untreated, good performance status patients, and docetaxel 75 mg/m^2^ and ifosfamide 2250 mg/m^2^ for previously treated patients. This schedule is worthy of further exploration in non-small cell lung cancer and upper gastro-intestinal malignancies.
